# The Application of Ultrasound in 3D Bio-Printing

**DOI:** 10.3390/molecules21050590

**Published:** 2016-05-05

**Authors:** Yufeng Zhou

**Affiliations:** Singapore Centre for 3D Printing (SC3DP), School of Mechanical and Aerospace Engineering, Nanyang Technological University, Singapore 639798, Singapore; yfzhou@ntu.edu.sg; Tel.: +65-6790-4482

**Keywords:** three-dimensional bio-printing, bioink, cell spheroids, ultrasound standing wave field, bioreactor, low-intensity ultrasound

## Abstract

Three-dimensional (3D) bioprinting is an emerging and promising technology in tissue engineering to construct tissues and organs for implantation. Alignment of self-assembly cell spheroids that are used as bioink could be very accurate after droplet ejection from bioprinter. Complex and heterogeneous tissue structures could be built using rapid additive manufacture technology and multiple cell lines. Effective vascularization in the engineered tissue samples is critical in any clinical application. In this review paper, the current technologies and processing steps (such as printing, preparation of bioink, cross-linking, tissue fusion and maturation) in 3D bio-printing are introduced, and their specifications are compared with each other. In addition, the application of ultrasound in this novel field is also introduced. Cells experience acoustic radiation force in ultrasound standing wave field (USWF) and then accumulate at the pressure node at low acoustic pressure. Formation of cell spheroids by this method is within minutes with uniform size and homogeneous cell distribution. Neovessel formation from USWF-induced endothelial cell spheroids is significant. Low-intensity ultrasound could enhance the proliferation and differentiation of stem cells. Its use is at low cost and compatible with current bioreactor. In summary, ultrasound application in 3D bio-printing may solve some challenges and enhance the outcomes.

## 1. Introduction

Since the first successful kidney transplant was performed in 1954 [1], organ transplantation has become a popular procedure for many incurable diseases. In the USA, about 79 patients undergo transplants daily. Because of the wide application of organ transplantation, there are obvious and acute needs for human organs (*i.e.*, heart, lungs, liver, kidney, and pancreas). However, the number of donors is limited, most mechanical devices are not reliable and xenotransplantation has inherent serious ethical issues. The waiting list for organ transplantation in the USA has more than 60,000 patients for kidney transplants, 3000 for heart, and 17,000 for liver, among who 17 will die every day according to data from the U.S. Department of Health and Human Services. This presents a burning problem and will become critical in the near future with increasing life expectancy. Furthermore, infections and immune rejection by the host are always serious for the transplantation [2]. Tissue engineering is an interdisciplinary field involving biological sciences and engineering to develop samples that restore, maintain, or enhance tissue function and repair injured or malfunctioning organs *in vivo* by mimicking native functional tissues and organs as a promising and permanent solution to the problem of organ failure [3,4,5,6]. In addition, tissue engineering has the potential for *in vitro* applications, such as the use of perfused human tissue for toxicological research, drug testing and screening, personalized medicine, disease pathogenesis, and cancer metastasis.

Classic tissue engineering uses a “top-down” approach, in which cells are seeded onto a solid biocompatible and biodegradable scaffold for growth and formation of their own extracellular matrix (ECM), representing a dominating conceptual framework or paradigm [7]. The main reasons of using the scaffold are to support the shape and rigidity of the engineered tissue and to provide a substrate for cell attachment and proliferation. Despite significant advances in the successful production of skin, cartilage, and avascular tissues *in vitro*, there are a number of challenges, such as fabricating larger, more complex, 3D functional tissues with high cell densities and metabolic activities [8,9,10]. Complete scaffold biodegradation is relatively slow, and tissue neomorphogenesis are laborious, expensive, and time-consuming. To implement effective vascularization of engineered samples to supply essential oxygen and nutrients after implantation the *in vitro* engineered tissue with established vascular network anastomoses with the host vasculature because of its much faster tissue perfusion than host dependent vascular ingrowth without compromising cell viability [11,12]. However, the problem of vascularization cannot be solved using biodegradable solid scaffolds because of its limited diffusion properties [13,14]. In addition, the lack of precise cell alignment, low cell density, use of organic solvents, insufficient interconnectivity, challenges in integrating the vascular network, controlling the pore distribution and dimensions, and manufacturing patient-specific implants are all major limitations in scaffold-based technology [15]. Microscale technologies used in biomedical and biological applications, such as 3D bio-printing, are powerful tools for addressing them, for example in prosthesis, implants [16,17], and scaffolds [18].

**Figure 1:**
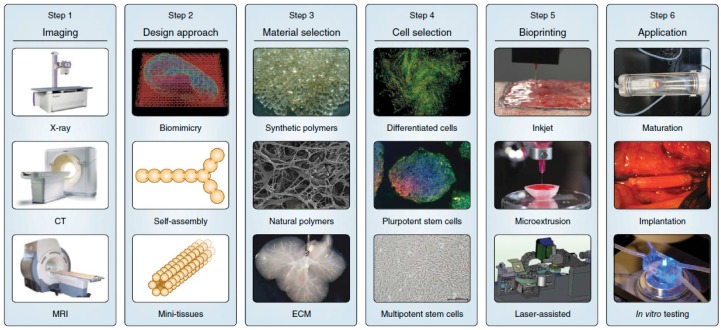
Typical six processes for 3D bioprinting: (**1**) imaging the damaged tissue and its environment to guide the design of bioprinted tissues/organs; (**2**) design approaches of biomimicry, tissue self-assembly and mini-tissue building blocks are sed singly and in combination; (**3**) the choice of materials (synthetic or natural polymers and decellularized ECM) and (**4**) cell source (allogeneic or autologous) is essential and specific to the tissue form and function; (**5**) bioprinting systems such as inkjet, microextrusion or laser-assisted printers; (**6**) tissue maturation in a bioreactor before transplantation or *in vitro* applications, courtesy of [24].

Three-dimensional printing was first introduced in 1986 [19], and now about 30,000 3D printers are sold worldwide every year. Recent advances in 3D bio-printing or the biomedical application of rapid prototyping have enabled precise positioning of biological materials, biochemicals, living cells, macrotissues, organ constructs, and supporting components (“bioink”) layer-by-layer in sprayed tissue fusion permissive hydrogels (“biopaper”) additively and robotically into complex 3D functional living tissues to fabricate 3D structures. This “bottom-up” solid scaffold-free automatic and biomimetic technology offers scalability, reproducibility, mass production of tissue engineered products with several cell types with high cell density and effective vascularization in large tissue constructs, even *in situ* organ biofabrication, which greatly relies on the principles of tissue self-assembly by mimicking natural morphogenesis [20]. The complex anatomy of the human body and its individual variances require the necessity of patient-specific, customized organ biofabrication [8,21,22]. Skin, bone, vascular grafts, tracheal splints, heart tissue, and cartilaginous specimen have already been printed successfully. Compared with conventional printing, 3D bio-printing has more complexities, including the selection of materials, cells, growth and differentiation factors, and challenges associated with the sensitive living cells, the tissue construction, the requirement of high throughput, and the reproduction of the micro-architecture of ECM components and multiple cell types based on the understanding of the arrangement of functional and supporting cells, gradients of soluble or insoluble factors, composition of the ECM, and the biological forces in the microenvironment. The whole process integrates technologies of fabrication, imaging, computer-aided robotics, biomaterials science, cell biology, biophysics, and medicine, and has three sequential steps: pre-processing (planning), processing (printing), and post-processing (tissue maturation) as shown in Figure 1 [23].

In this paper, available technologies and trends of 3D bio-printing in tissue engineering, especially preparing cell spheroids as bioink, printing bioink into complex structure and composition, crosslinking, tissue fusion, and tissue maturation with effect vascularization, are reviewed. The technical challenges and limitations found in recent studies are discussed. In addition, the application of ultrasound in this emerging field is also introduced. The cell spheroids could be formed in ultrasound standing stand field in a short time (within minutes) with high cell density and viability. The proliferation and differentiation of stem cells are enhanced using low-intensity ultrasound. Altogether, the use of ultrasound technology in 3D bio-printing may enhance the outcome.

## 2. 3D Bioprinting

Three dimensional bio-printing recently has made significant progress towards practice, such as representing the complexity of natural ECM and reconstituting the intrinsic cellular morphologies and functions [25,26]. The first step of 3D bio-printing is a comprehension of the complex, heterogeneous architecture of tissues and organs. CT and MRI with high resolution and contrast usually work collaboratively with computer-aided design and computer-aided manufacturing (CAD-CAM) toolbox and mathematical modeling to collect the tomographic and architectural information on 3D cellular, tissue, organ, and organism structure and function. Reconstruction can be viewed as contour stacks, wire-frame, shaded, or solid models with adjustable lighting, transparency, and reflectivity [24]. To accurately reproduce the tissue or organ, 2D cross-sections can be used directly for layer-by-layer deposition. Furthermore, computer modeling can also predict mechanical and biochemical properties of fabricated constructs.

**Figure 2:**
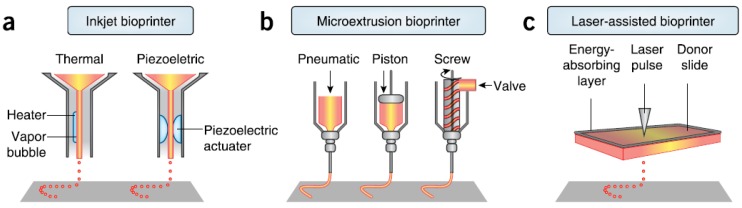
Schematic diagram of (**a**) thermal inkjet printers electrically heating the printhead to produce air-pressure pulses that force droplets from the nozzle, whereas acoustic printers using pulses formed by ultrasound pressure from piezoelectric element; (**b**) microextrusion printers using pneumatic or mechanical (piston or screw) dispensing systems to extrude bioink beads; and (**c**) laser-assisted printers utilizing focused laser beams on an absorbing substrate to propel bioink onto a collector substrate, courtesy of [24].

There are three major technologies used for depositing and patterning biological materials as shown in Figure 2: inkjet [27], microextrusion [22,28], and laser-assisted printing [29]. Inkjet (drop-on-demand or continuous ejection) printers are the most commonly used at low cost, high resolution, high speed, and wide availability due to their simple components and ready-to-use design and control software [30]. Drops from the cartridge are ejected onto a substrate under the control of an electronic elevator stage [31,32]. The size of droplets and the rate of ejection could be adjusted by controlling the pulse, duration, and excitation amplitude [33]. It is also possible to produce an inhomogenous distribution of cells, materials, and growth factors [34]. The small amount of material used in inkjet printing and its low reagent costs are its advantages. However, the shear stress imposed on cells at the nozzle tip may induce damage to the cell membrane and lysis [27]. Other limitations and challenges include low material viscosity, excessive force required for drop ejection [35], inability of reproducing droplets, low cell densities in liquid, clogging of the nozzle orifice, cell aggregation and sedimentation in the cartridge reservoir, and the limited number of simultaneous fluid ejection [33,36]. Adding surfactants to improve the reliability of droplet formation and frequent stirring of the cell mixture to prevent sedimentation may damage cells.

Microextrusion printers are the most common and affordable ones in tissue and organ engineering research, and are driven by pneumatic or mechanical (piston or screw) dispensing systems [37,38]. Pneumatically driven printers have simple driving mechanisms with the force limited only by the air-pressure capabilities, but a delay in output. Mechanically driven systems provide more direct control over the material flow and have greater spatial control of smaller and more complex components, especially for high-viscosity materials, but with reduced maximum force capabilities. The major advantage of microextrusion bio-printing technology is the very high cell densities achievable in the deposition. However, cell viability after microextrusion (40%–86%) decreases with extrusion pressure and speed, nozzle gauge, and the viscosity of hydrogels, and is lower than that of inkjet bio-printing because of the shear stresses applied to cells in viscous fluids [39]. Increasing microextrusion resolution and speed and development of parallel process are its current challenges.

Laser-assisted bio-printing (LAB) consists of a high power laser pulse, a focusing optics system, a “ribbon” (e.g., glass) covered with optically-absorbing (e.g., gold or titanium) and biological material layers, and a collecting substrate. The laser-induced bubbles produce shock waves to transfer cells toward the collector substrate. LAB systems are nozzle-free, with none of the clogging problems of other printing technologies. The technique has been successfully applied to peptides, DNA, and cells with negligible effect on cell viability and function using a laser pulse repetition rate of 5 kHz [29,40,41,42,43]. However, preparation of ribbons is time-consuming and even more onerous for co-deposition of multiple cell types and materials. The gravitational and random setting of cells in the precursor solution, prolonged fabrication time, limited printing capability in the third dimension, and the requirement for photocrosslinkable biomaterials are its shortcomings. A comparison of these three types of bioprinters is provided in Table 1 [39,42,44].

## 3. Bioink

### 3.1. Bioink and Its Preparation

Inclusion of cells within biomaterials to prepare bioink is the cornerstone of 3D bio-printing and should fulfill not only the biological requirements for cells but also the physical and mechanical ones of the printing process [45]. The common bioink in use is a biomimic, autonomous self-assembly, micro-tissue with similar cell density as normal organs or tissues. The cells used for bio-printing should survive the microextrusion process and bear physical forces (*i.e.*, shear stress and pressure) and biological stressors (*i.e.*, toxins, enzymes, and nonphysiological pH) once implanted. The properties of bioink depend on cell phenotype, culture medium, growth factors, culture conditions (*i.e.*, static or dynamic), cell aggregation methods, and tissue maturation. The preparation of bioink requires scalability in fabrication, maximally standardized in the size, no significant cell injury, DNA damage, and clogging problem during biofabrication, no compromising on the aggregates’ capacity for sequential tissue fusion, and flexible enough for a diversity of aggregates with complex composite structure. Functional micro-tissues self-assembly of precisely aligned tissue spheroids with “built in” intraorgan branched vascular system but no solid scaffolds can be used to fabricate functional macro-tissues and organs after accelerated tissue maturation; weak material properties are required for efficient biofabrication, and viability and shape of solid scaffold-free 3D tissue constructs should be maintained by crosslinking.

**Figure 3:**
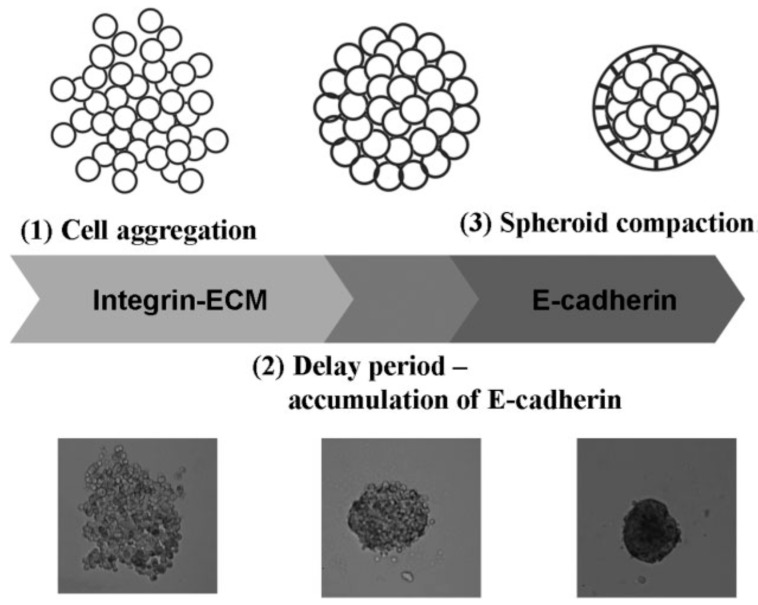
The processes of the spheroid formation: (**1**) formation of loose cell aggregates via integrin-ECM binding; (**2**) a delay period for cadherin expression and accumulation; (**3**) formation of compact spheroids through hemophilic cadherin-cadherin interactions, courtesy of [46].

Autonomous and individual cells organizing into multicellular units and producing the final tissue construct without human intervention is called self-assembly [47,48,49]. It is shown that biological tissues can be engineered with specific compositions and shapes by exploiting cell-cell adhesion and ECM growth of cultured cells, thereby mediating inflammatory responses. The cells in suspension or in a nonadhesive environment (*i.e.*, agarose, Primaria dish, and substrates without cellular attachment molecules) aggregate slowly and then show enhanced viability and functionality [50]. Tissue spheroids are considered as “voxels” in printing at desirable size fabricated easily at large scale as shown in Figure 3. The diameter of the spheroid is 200–400 μm due to the constraints of diffusion of 150–200 μm to many molecules. Therefore, the dense core of spheroids and inefficient mass transport often result in morphological disintegration, such as cell death owing to the lack of oxygen and nutrient supply and production of metabolic waste [51,52,53,54]. The cell organization leads to an increase in cell-mediated collagen gel contraction and collagen fibril reorganization with a homogenous cell distribution, indicating enhanced cell-mediated collagen matrix remodeling. Many mammalian primary or progenitor cells can aggregate and differentiate into 3D multicellular spheroids (MCSs) which are more heterogeneous in metabolic state and gene expression than monolayer cells. MCSs in a size above 500 μm are analogous to structural and functional properties of avascular tissue or tumor mass in layered structures, a necrotic core with quiescent cells surrounded by a viable rim with proliferating cells [46].

Conventional methods of producing mature multicellular pellets and spheroids take several days (or even weeks) and need manual selection to achieve a homogeneous population [55]. Cells aggregate in spherical shapes to maximize intercellular adhesion and minimize intercellular energy [56]. Several alternatives techniques have been developed [57]. Micropatterning of cell-adhesive contacts using ECM proteins coated onto microfabricated stamps by photolithography or microcontact printing is used to adhere cells into predesigned patterns. The pellet culture technique uses centrifugal force (*i.e.*, 500 G for 5 min) to concentrate cells at the bottom of a tube to enhance cell-to-cell adhesions. The spinner culture method creates spheroids by preventing cells in suspension from settling and promoting cell-to-cell collisions via constant stirring using the generated convective forces [57]. Owing to shear forces this method is not useful for cells with low cohesiveness and high sensitivity, or for adherent cells that undergo apoptosis. A single spheroid is formed at the bottom of the drop in a small volume (20–30 μL) of a cell suspension utilizing hanging drop technique [58]. Spheroid size and cellular composition are controlled by adjusting the cell density in each drop, up to 384 spheroids in a single array [59]. Rotating cell containers (15–25 rpms) create microgravity for spheroid formation. Multi-cellular cell sheets are produced by culturing cells on a polymer and then releasing them for further incubation on a nonadhesive surface where they will compact and form spheroids. Cell sheets could be wrapped around a mandrel to create a tube and a thicker tissue. Cells are flowed through a microchannel into micro-chambers where they are partitioned and exposed to micro-rotational flow for cells aggregation [60]. Recent advances in digital (droplet-based) microfluidics allow fabricate thousand tissue spheroids with complex structure and composition in seconds [61]. Micro-molded nonadhesive hydrogels on an array of cylindrical pegs and rapid prototyping could form up to 822 spheroids in a single mold with homogenous shape, size, and cell composition. External forces are also applied in forming bioink. In electric fields, positive dielectrophoresis in a low conductivity iso-osmotic solution is used to concentrate cells [62]; whereas cells are incubated with magnetic cationic liposomes containing a magnetite core (Fe_3_O_4_) in magnetic fields [63,64]. Cell adhesion is very nonspecific, and it is difficult to control spheroid size. The physiological changes to the cells caused by these external forces are not well characterized.

### 3.2. Preparation Using Ultrasound Approach

**Figure 4:**
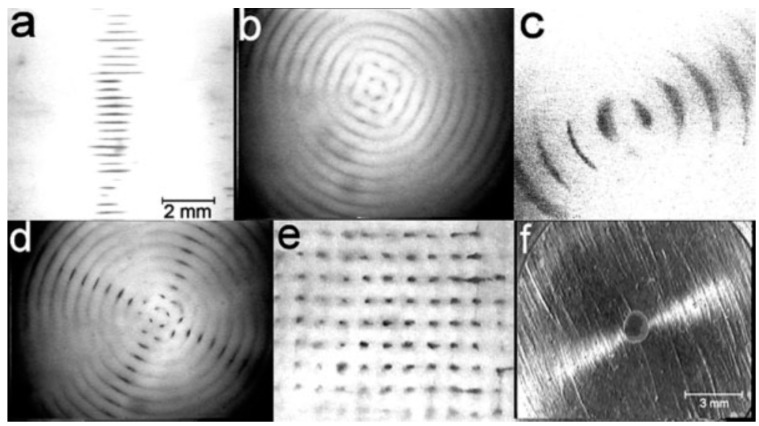
Cell aggregation under ultrasound standing wave and view normal to sound propagation (**a**) column of discoid aggregates in a plane cylindrical resonator; (**b**) initial pattern in tubular resonator-concentric cylinders; (**c**) aggregates of different sizes in a tubular resonator; (**d**) in plane and tubular resonator; (**e**) in a pair of two transducers perpendicular to each other; and (**f**) view in the direction of sound propagation of 3D RBC aggregate in the center of half-wavelength resonator, courtesy of [71].

The micro-particles or suspended cells experience an axial direct acoustic radiation force (DRF) in an ultrasound standing wave field (USWF) without any prior modification of the cell surface as shown in Figure 4 [65,66]. USWF has a periodic presence of acoustic pressure nodes (no vibration) and anti-nodes (maximum vibration) that have half-wavelength intervals perpendicular to the sound propagating direction [67,68,69]. The axial component of DRF drives particles towards the pressure node planes, whereas its lateral components (two orders of magnitude smaller) concentrate particles/cells laterally into clumps within the planes [70]. The DRF is described as:

(1) $$F_{rad}=\left(\frac{-\pi P_{0}^{2}V\beta_{0}}{2\lambda }\right)\cdot \phi \cdot \sin\left(\frac{4\pi z}{\lambda }\right)$$

where *P*_0_ is the acoustic pressure in USWF, *V* is the spherical particle volume, λ is the acoustic wavelength, *z* is the axial distance from pressure nodal planes, and φ is a contrast factor:

(2) $$\phi =\frac{5\rho_{p}-2\rho_{0}}{2\rho_{p}+\rho_{0}}-\frac{\beta_{p}}{\beta_{0}},$$

where ρ*_p_* and ρ_0_ are the density of the particle and fluid, respectively, and β*_p_* and β_0_ is its corresponding compressibility. Eckart streaming may present in the volume of all multi-wavelength resonators, and cause the adhesion of some single cells and small cell clusters in the chamber to the aggregates continuously during sonication [71]. Meanwhile, the large drag sweeps single particles away or limits the size of a growing aggregate by drawing particles off its perimeter.

**Figure 5:**
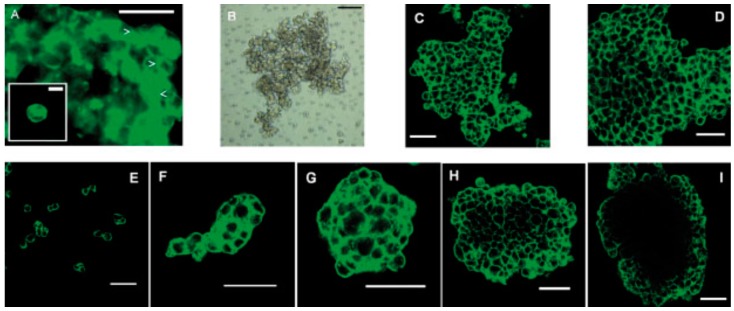
(**A**) Epifluorescence micrograph of 3D HepG2 aggregates by ultrasound standing wave and F-actin stained with Phalloidin-Alexa 488 with junctional F-actin marked with arrowheads (bar 25 μm) and an unsonicated single cell shown in inset (bar 10 μm); (**B**) aggregate maintained on a P-HEMA-coated surface after 1 day under light microscope, confocal micrograph of F-actin staining after 1 day (**C**) and 18 days (**D**); and confocal micrograph of F-actin staining in gyrotatory-produced aggregates after (**E**) 2 h; (**F**) 1 day; (**G**) 3 days; (**H**) 9 days and (**I**) 18 days (**B**–**I**, bar 50 μm), courtesy of [77].

Controlling cell patterning, cell function, and ECM organization are primary challenges to successfully fabricate functional tissues and organs *in vitro*, which may be addressed by the use of USWF [72]. Uniformly shaped and sized aggregates are fully formed within 2 min of ultrasonic exposure (the acoustic pressure of 0.85 MPa for the first 1 min for aggregation followed by 0.09 MPa sonication in the remaining 4 min to levitate aggregate in the trap), and no necrotic core (a sign of hypoxia) is observed in the aggregates 1 day after preparation [73]. The aggregates allow a better diffusion of oxygen and nutrients to the core. The size of the USWF-induced aggregates in the size of 0.4–2.6 mm is dependent on the cell concentration (10^4^–5 × 10^6^/mL) and acoustic pressure. Cells in non- and encapsulated 3D HepG2 aggregates have viability of 70%–80% over 10 days in culture, increased proliferation (doubled cell number) and the thickness of aggregate, while encapsulated aggregates secrete 4.5 times higher albumin levels than non-encapsulated ones [74]. The aggregates are also mechanically robust and preserve their core structure after removal from the resonator as observed in surface electron microscopy and confocal microscopy as shown in Figure 5. Furthermore, USWF can also co-locate active or inactive cell-bound molecules with cell aggregates, such as the ECM protein (*i.e.*, fibronectin) [75]. Cell aggregates induced in USWF accelerate the formation and elongation of sprouts, promote collagen fiber alignment, and mature endothelial cell sprouts into lumen-containing anastomosing vascular tree-like networks that branch into small capillary-sized structures. However, sprout formation is delayed in sham-exposed and absent from sham collagen gels, respectively. USWF technology leads to rapid and extensive vascularization and, therefore, provide a new strategy to vascularize engineered tissues *in vitro* [76]. In summary, ultrasound-formed encapsulated high-density cell aggregates has technical advantages of 3D structure, rapid formation, mechanical stability, and very low hypoxia at the core as well as good functionality of specific biomarkers (*i.e.*, CYP450-1A1 and CYP450-3A4, glutathione-*S*-transferase expression, C-18 expression, glucose, and lactate release for HepG2).

### 3.3. Bioink Characterization

The properties of rounded micro-tissues can be measured by tensiometry or classic tensile tests using two parallel plates [20]. Incorporation of tissue spheroids with magnetic or fluorescent microbeads is used to characterize the material properties of cell spheroids no matter of tissue fusion as well as to non-destructively monitor tissue maturation. The fluorescent recovery after photobeaching (FRAP) method is used to determine the density of ECM molecules [78]. The measurement of electroconductivity and electric impedance is another non-invasive characterization approach [79].

### 3.4. Medium of Bioink

Since the initially engineered construct is quite fragile, the nascent tissue structure requires some form of transient non-adherent support with a cell-inert substrate that provides mechanical buttressing without affecting the cellular biology and can be easily removed after the tissue fusion to leave an intact construct. Tissues and organs are formed without solid scaffolds as embryonic development. Biocompatible, nontoxic, and dispensable biosupports should solidify rapidly and be functional with growth factors for high cell attachment, proliferation, differentiation, and viability. Naturally derived hydrogels and polymers (*i.e.*, collagen, hyaluronic acid, alginate, fibrin, gelatin, and hyaluronan) are used as substrates for various stem cells (*i.e.*, human embryonic, bone marrow, and adipose-derived stem cells) as encapsulation because of their similarity to human ECM and inherent bioactivity [80]. In comparison, both hydrophilic and absorbent synthetic hydrogels and polymers (*i.e.*, photocured acrylates, polyurethane foam, galactosylated nanofiber meshes, and poly-l-lactic acid matrices) can be tailored to specific physical properties for printing, but have poor biocompatibility, toxic degradation, and reduced mechanical properties [46]. Materials with higher viscosity provide structural support for the engineered tissue while cell viability and function would be maintained in those with lower viscosity. Non-Newtonian materials with shear-thinning properties that decrease the viscosity in response to the increased shear rate are commonly used for microextrusion. Although the viscosity of hydrogel could be increased at low temperature for structural support after 3D printing, cellular viability will be decreased. Increasing the concentration of polymer could improve the printability. However, some large molecular-weight polymers are broken down into oligomers or monomers and then cause inflammation and other detrimental effects.

## 4. Crosslinking

After printing and deposition, crosslinking gelation is usually initiated by physical and/or chemical process, such as pH, thermal transitions, or ultraviolet illumination, but often slows the bio-printing process. Physical crosslinking is a reversible interaction, depending on the meshes of polymer chains, ionic interactions, and hydrogen bridges, so that it is biologically compatible with growth factors and living cells [81]. Poor mechanical properties are the major drawback of the physical reaction. Thus, post-processing crosslinking and an additional agent is usually needed. However, chemical crosslinking forms new covalent bonds, which have relatively high mechanical stability. The reaction of their functional groups (*i.e.*, OH, COOH, and NH_2_) of natural and synthetic polymers with crosslinkers such as aldehyde (*i.e.*, glutaraldehyde, adipic acid dihydrazide) is also used for crosslinking. For example, CaCl_2_ (3%, *w*/*v*) is added to crosslink alginate. However, this type of crosslinking may chemically modify the properties of ECM materials, and sometimes decrease viability and functionality of cells [82], and generate small mesh networks, which limits the mobility and migration of encapsulated cells [83].

## 5. Tissue Fusion

**Figure 6:**
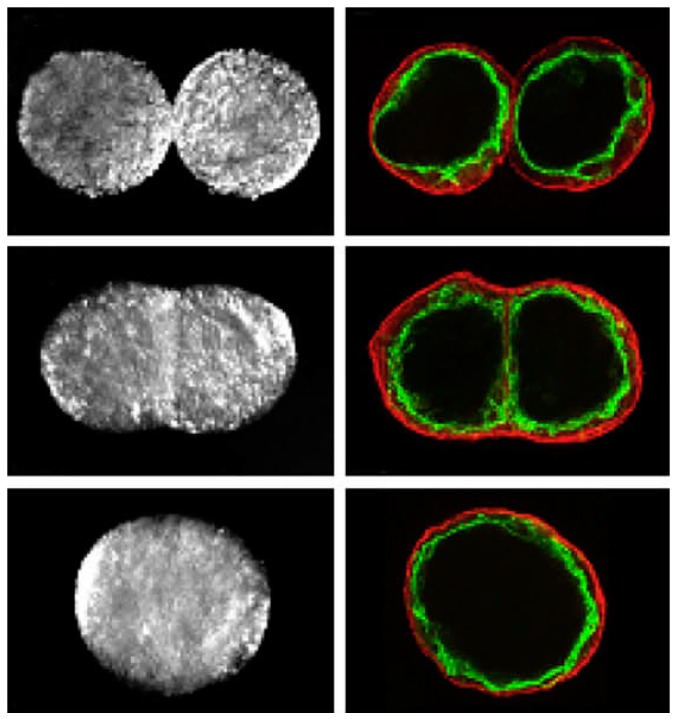
Bioprinting of segments of intraorgan branched vascular tree using uni-lumenal vascular tissue spheroids in hanging drop after tissue fusion, courtesy of [20].

After bio-printing, closely placed tissue spheroids undergo tissue fusion as shown in Figure 6, similar to embryonic development [84]. The kinetics of tissue fusion of two rounded embryonic heart cushion tissue explants fits quite well that for two fluid drops [47]. Moreover, tissue spheroids are indeed fluidic structures based on directly measured surface tension and calculated viscosity [47].

The ability of the multicellular aggregates to fuse is the molecular consequence of tissue liquidity. Therefore, tissue fusion is an essential phenomenon of fluid mechanics determined by surface tension forces which is described by Steinberg’s differential adhesion hypothesis (DAH) [85,86,87]. DAH posits that cell types self-segregate due to differences in cell-to-cell adhesion or apparent surface tension, with those cells of highest cohesion (like-to-like adhesion) sorting to the inside of a spheroid and those cells with lower cohesion sorting to the outside. The distinct cell adhesion increases the surface tensions of cohesive tissues [85]. The tissue interfacial tensions and viscosities measured from varieties of cells are found consistent with the mutual sorting behavior of the corresponding tissues. The strong agreement between experiment and model suggests that tissue liquidity is indeed the morphogenetic mechanism underlying post-printing structure formation. Meanwhile, motile living cells, cytoskeleton, and the number, redistribution, and activation of cell adhesion receptors are important for the tissue fusion [88,89]. The accumulated ECM, restricted cell motility, and enhanced tissue cohesion can change kinetics or impede the tissue fusion [90]. Differential interfacial tension hypothesis (DITH) factors in contributions from cytoskeletal components and cell adhesion molecules. Therefore, the effect of accumulated ECM and its specific molecules as well as ECM remodeling on tissue spheroids’ material properties and tissue fusion remains to be elucidated. The rapid increase in biomechanical integrity of the engineered construct during perfusion is mostly due to extensive de-novo ECM deposition [91].

## 6. Bioreactors

### 6.1. Post-Processing in 3D Bioprinting

Post-processing is the most essential and critical step in bio-printing, and the development of bioreactors and post-processing technologies for effective and accelerated tissue maturation as well as non-invasive and non-destructive biomonitoring is in great need. Bioreactors can maintain the cell viability of engineered constructs and reduce the time necessary for tissue fusion, remodeling, and maturation in combination with factors that promote angiogenesis and innervation [92] and maintain or preserve cell viability. At this stage, a controlled microenvironment in a bioreactor is required for temperature, buffering, oxygenation, pH, nutrient and gas concentration, sterility, delivery of trophic factors as well as regulation of specific mechanical stimulation [93]. Cells proliferate progressively in printed cell-hydrogel construct in an initial low density [94,95,96]. Collagen and elastin are two most important ECM proteins in the human connective tissues as well as stromal elements of parenchymal organs so that tissue maturation essentially is to enhance collagen and elastin deposition. With native tissue remodeling, the cell-cell and cell-ECM interactions form a complex network of important biochemical and biomechanical signals for normal cell physiology, mediating cell adhesion, controlling cell function, and guiding tissue development. In the solid scaffold-free printed 3D macro-tissue constructs, rapid tissue maturation or fluid to solid transition is required to maintain their shape, composition, and integrity. The sacrificial materials can provide the required structural and mechanical properties, either during the printing to allow sufficient crosslinking in the construct [97,98] or incorporation into the structure until the endogenously produced materials can perform their function. As a scaffold degrades, the embedded cells secrete proteases and produce ECM proteins to define the new tissue. The degradation kinetics of the scaffold with appropriate functional and mechanical characteristics should be controlled ideally (but challenging) to match the cellular ability of the replaced materials with their own ECM proteins upon degradation. In addition, degradation byproducts should be biocompatible.

Controlling the release of vascular endothelial growth factor (VEGF), an angiogenic factor for vascularization of the printed constructs, is an important but unresolved problem in tissue engineering [99,100]. The response of aggregated endothelial cells to the proangiogenic factors is limited in the absence of cell-cell contacts, subsequently preventing apoptosis [101]. When activating fibroblasts in a collagen gel with platelet-derived growth factor (PDGF), gel volume reduction of more than 70% occurs. However, when fibroblasts are encapsulated in a crosslinked ECM containing unmodified collagen, there is no contraction. The genetic approach by viral transfection or use of small molecules, such as (*i.e.*, vitamin C, lysyl oxidase, TGFβ) or non-enzymatic glycation with ribose, to induce cell proliferation and prevent senescence may solve this problem [102]. However, use of TGFβ generates undesired tissues different from the native cartilages and suffers from hypertrophic changes at late stages of differentiation both *in vitro* and *in vivo* [103,104]. Thus, its role is not always definite in tissue engineering, such as chondrogenic differentiation of mesenchymal stem cells (MSCs). The inhibition of versican synthesis by antisense DNA accelerates the formation of elastic fibers [105]. Embryonic cushion tissue explants transfected with the periostin gene have improved biomechanical properties, showing a direct effect of genetic manipulations on tissue structural integrity [106]. Finally, the introduction of fibroblasts into smooth muscle aggregates can accelerate vascular tissue maturation. The enzymatic disaggregation of spheroids and the direct counting of cells are used to quantify spheroid growth and the response to growth factors. To determine where cell proliferation occurs, spheroids have been sectioned or visualized via confocal microscopy and immunostained for standard proliferation markers such as Ki-67 or bromodeoxyuridine labels. Mechanical conditioning, such as under pulsatile flow, can improve vascular tissue maturation. In the physical stimulation with appropriate pressure and associated shear stress, vascular tissue maturation induces factors for perfusion. However, the biomechanical properties of such vascular constructs are inferior to true blood vessels.

In order to produce effective vascularization in thick engineered tissues, an intimate knowledge of the tissue genesis and organogenesis in embryonic development as well as the capability of manipulating the environment to drive embryonic mechanism is usually used as a guide. Organization and branching patterns of an intraorgan vascular tree “built-in” 3D macro-tissue must be organo- and vaso-specific. The arteries and veins at the onset of an intraorgan vascular tree should connect with recipient large vessels and microvascular network, respectively. A pre-vascular network was found within 10 days in mixed spheroids of human MSCs and human umbilical vein endothelial cells (HUVECs) [107,108]. Vascular tissue spheroids, mono-lumenized vascular spheroids, and histotypical microvascularized tissue spheroids could form a large vascularized tissue through the apoptosis of polarized central cells and integrate with the host vascular system after implantation [109]. Small isolated fragments of the microvascular networks can be reunited by self-assembles either from single cells placed into hydrogel [110] or from endothelial cell spheroids [109,111]. Thus, it is technologically feasible to build a intraorgan branched vascular tree with 10–12 orders of branching [20].

### 6.2. Vascularization of USWF-Induced Endothelial Cell Spheroids

**Figure 7:**
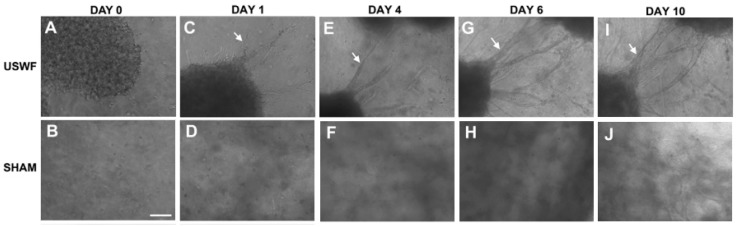
Phase-contrast images of neovessel formation and sprout in suspended endothelial cells in an unpolymerized collagen type-I solution following ultrasound standing wave field (USWF) exposure (**A**,**C**,**E**,**G**,**I**) shown by white arrow and sham-exposed (**B**,**D**,**F**,**H**,**J**) in scale bar of 100 μm, courtesy of [76].

**Figure 8:**
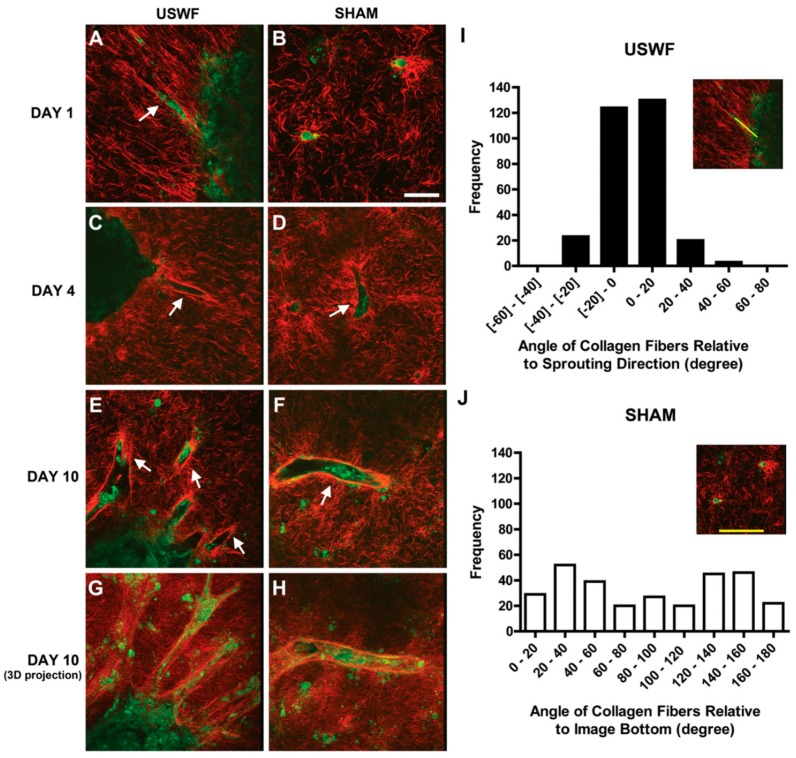
Intrinsic cellular autofluorescence in second-harmonic generation microscopy of endothelial cell bands (green) suspended in unpolymerized collagen type-I fibers (red) on (**A**,**B**) day 1; (**C**,**D**) day 4; and (**E**,**F**) day 10 incubation treated by ultrasound standing wave field (USWF) or sham-exposure, arrows show endothelial cell sprouts in scale bar of 50 μm, and the histograms of the occurrence frequency of collagen fiber angles in (**I**) USWF- and (**J**) sham-exposed constructs. collagen gels, courtesy of [76].

USWF-induced discoid of endothelial cells can initiate a cascade of cell migration, proliferation, and ECM remodeling for neovessel formation as shown in Figure 7 and Figure 8 [112]. One day after preparation, USWF induced cell discoid clearly show multiple endothelial cell sprouts originating from it. On day 4, such sprouts increase in length with the visible formation of branches and interconnections between them. Although the elongated cells in the control group (sham-exposed) persist on day 6 and 10 and exhibit some intercellular connections, vascularization of the USWF-exposed group is significant. USWF-induced endothelial cell could produce viable, anastomosing, capillary-like networks, both neighboring sprouts and adjacent cell discoids throughout the 3D construct with large arteriole-sized lumen branching into capillary-containing structures on day 6, which progress into longer and thicker structures on day 10. The proliferation of USWF-induced cell discoids is observed with emerging sprouts. At the onset of capillary sprout formation, elongated and mediated reorganization of ECM collagen into aligned fibrils is in the direction of sprout outgrowth as the natural capillary sprouting. In the control group, collagen fibers extend into the collagen matrix well beyond the tip of the sprout, but are organized randomly throughout the collagen gel [113].

### 6.3. Effect of LIUS on Tissue Maturation

**Figure 9:**
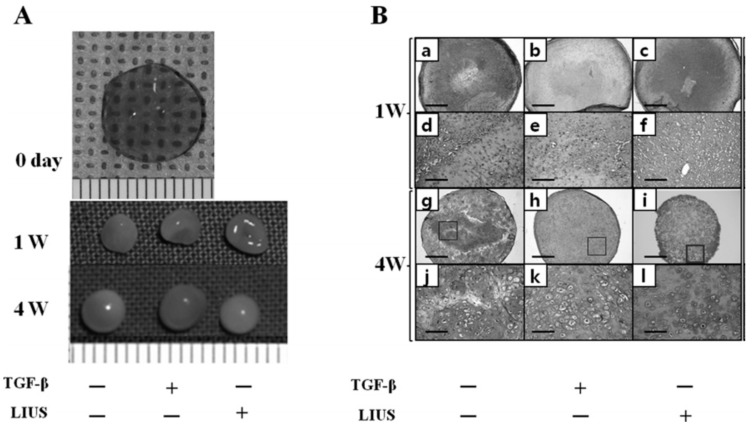
Effect of low-intensity ultrasound (LIUS) on the chondrogenesis of rabbit MSCs seeded in fibrin-HA and cultured in chondrogenic-defined medium untreated, treated with TGF-β3 (10 ng/mL), or treated with LIUS (100 mW/cm^2^) for 1 and 4 weeks, (**A**) the gross images of the constructs at 0, 1, and 4 weeks in scale bar of 1 mm; and (**B**) images of safranin-O/fast green staining in scale bar of 1 mm for (**a**–**c**,**g**–**i**), 200 mm for (**d**–**f**,**j**–**l**), courtesy of [120].

The lack of methods required to fully and effectively differentiate stem cells has been a major obstacle for cell therapy in tissue engineering. Chondrogenic differentiation and cartilage tissue formation derived from MSCs requires a 3D environment and are highly dependent on both biological and mechanical factors. The introduction of fibrin-hyaluronic acid (HA) shows more accumulation of sulfated glycosaminoglycans (GAGs) and high efficiency in promoting chondrogenic differentiation and cartilage matrix synthesis of MSCs *in vitro* than the alginate group. Mechanical stimulation, such as cyclic compressive loading produced by low-intensity ultrasound (LIUS), can not only induce chondrogenic differentiation in MSCs [114,115], but also enhance the viability of MSCs, increase the integrity of the differentiated tissues, and delay hypertrophic changes during differentiation as shown in Figure 9 [116]. LIUS is simple and cost-effective to activate chondrocyte phenotypes *in vitro* [117,118] and improve cartilage repair in animal models [119]. LIUS further enhances chondrogenesis of MSCs cultured in fibrin-HA *in vitro* to construct high-quality cartilage tissues [120]. The combination of TGF-β3 and LIUS shows an increase in collagen accumulation and compressive strength at week 4, and the LIUS alone shows more collagen than the only use of TGF-β3. However, TGFβ alone shows no significant effect on chondrogenic differentiation and calcification of the implant in nude mice. LIUS increases the synthesis of endogenous TGFβ in MSCs or the access of exogenous TGFβ to the tissue center meanwhile LIUS has consistent *in vitro* and *in vivo* effect without exogenous TGFβ in various experimental conditions [121,122].

The biochemical effect of LIUS is demonstrated by the expression of proteoglycans and type II collagen using RT-PCR and chemical assays. LIUS stimulation increases the expression of proteoglycans and collagens in the construct, such as tissue inhibitor of metalloproteinases-2 (MMP-2), but shows no effect on MMP-3 or mRNA levels of MMP-13, and type I and X collagens [121]. Thus, LIUS may inhibit degradation of ECM proteins and hypertrophy of differentiated MSCs. LIUS for a week could program MSCs to differentiate well into chondrogenic lineages at a high proliferation rate or to better maintain chondrogenic phenotypes during subsequent culture for a long time [121]. Since TGFβ is not sufficient to induce chondrogenic differentiation of human MSCs, BMP-1 or BMP-2 is necessary. LIUS may use different cellular surface receptors and signaling pathways from those for TGFβ (*i.e.*, Smad), such as the mechanotransduction pathway including integrins, stretch‑activated ion channels, and interleukin‑4 [123,124]. The mechanism of inhibiting hypertrophy of cells by LIUS is not fully understood but different from that of enhancing chondrogenic differentiation because hypertrophic change occurs prematurely and often precedes the chondrogenic differentiation of MSCs [103]. Therefore, LIUS-induced inhibition of hypertrophy may be a prerequisite for the efficient chondrogenic differentiation of MSCs rather than its outcome. LIUS can reduce the expression of type X collagen in the core of the cartilage explants with no chondrogenic differentiation. However, there are no significant differences in MSCs differentiation in a PGA scaffold [125]. Chondrogenic differentiation of 3D MSCs in the use of TGFβ is cytotoxic and induces apoptosis. LIUS exposure could inhibit apoptotic events, reducing apoptosis-related genes (*i.e.*, p53 and bax) and inducing anti-apoptotic genes (*i.e.*, bcl-2 and PCNA) [121]. Overall, LIUS treatment can enhance the viability of MSCs by inhibiting cell apoptosis, regulate expression of genes involved in the integrity of the differentiated construct, and delay hypertrophy, and it is easy to apply in tissue engineering with reduced risk of tissue contamination [117]. Thus, sonication from outside of the incubator is possible.

## 7. Discussion and Summary

3D bio-printing is a promising and attractive technology for the fabrication of tissues and organs with complicated structure and function but no reliance on endogenous host regenerative capacity. It has achieved great progress and breakthroughs in the last decade [91]. The design and development of a fully integrated human tissues and organs for industrial scale automated biofabrication lines using micro-tissues is imperative on the path to commercialization by optimizing the compatible technologies and integrating them seamlessly before developing something entirely new [126]. More complicated organ biofabrication lines may include a clinical cell sorter, stem cell bioreactor, cell differentiator, tissue spheroids fabricator and encapsulator, robotic bioprinter, and perfusion bioreactor. However, bio-printing technology is still in its infancy, and there are some technological challenges to be addressed for success: standardized large-scale fabrication of uniform tissue spheroids with the increased diversity of bio-processbility and functionalities in high cell density but less cytotoxicity; development of continuous and digital industrial bioprinters with improved nozzle and cartridge design for compactness, affordability, resolution, repeatability, motion capability with high degree-of-freedom, motion speed, cell viability, sterilibility, versatility, biocompatibility, and user friendly interface; use of MCSs with different viscosities, long-term functionality, and multiscale hybrid bio-printing processes; use of hydrogel or polymer with appropriate solidification speed; robotic 3D bio-printing of organ constructs; biofabrication of a intraorgan branched vascular tree in the macro-tissues for occlusion- or leak-free perfusion; non-destructive real-time monitoring using sophisticated embedded sensors and an automatic quality control system; and development of effective and low-cost bioreactor for accelerated tissue maturation [20,126]. However, the increased complexity and functionality of bioprinters in a single system can increase the failure chance. Close collaboration between biologists and engineers and the use of mathematical modeling to simulate tissue printing processes will significantly enhance, optimize, and accelerate the process [126].

Appropriate design of bioink should be explored further to meet the requirement of truly accelerated tissue maturation [127], such as hybrid tissue spheroids with rigid internal micro-scaffolds or biodegradable porous microcarriers with a rapid transition from fluid to solid state as a function of density as a tradeoff between the solid scaffold and micro-tissue approaches. In addition, nano-assembly and self-assembly of ECM molecules are also emerging methods [128]. A practical concern of translation is that it is significantly more difficult to manipulate, culture, and bio-print human cells than rodent or pig cells. Furthermore, primary cells from old human donors have significantly biochemical and epigenetic changes that make them less likely to easily recapitulate developmental processes *in vitro* [91].

2D cell culture does not faithfully replicate all of the mechanical and biochemical signals *in vivo* because of cell-to-plastic interactions prevailing rather than the crucial cell-to-cell and cell-to-ECM interactions for normal cell function, which simultaneously deliver mechanical, biochemical, and electrical signals that can influence cell shape, motility, proliferation, and differentiation as well as gene expression [129,130]. The additional dimension of 3D constructs results in different cell activities, including morphology, proliferation, and gene and protein expression [131]. Gradients of soluble components are established due to the barriers to diffusion (e.g., cellular compaction, gap junctions, and cellular efflux systems) imposed by the spheroid as well as the consumption rates and production of these factors by the cells [132,133]. Therefore, MCSs with different cells phonotypes have characteristics of avascular tumor nodules and heterogeneous tumor microenvironment and are used as an *in vitro* cost-effective high-throughput platform for anti-tumor drug toxicity screening and pharmaceutical development rather than using a large number of conventional expensive animals [134,135]. Due to the limited diffusion, thick tissues with high cell densities demand a functional vascular network to keep the cells alive [57]. Cancer cells (e.g., Hela and ovarian cancer cell lines) spheroids have a higher proliferation rate, MMP protein expression, and chemoresistance than those 2D construct [136,137]. Preclinical tumor models are often used to mimic physiological environments for tumor genesis and anti-cancer drug screening without ethical and safety limitations [138]. In addition, animal models established in immunocompromised mice often shows false effects on tumor growth [139]. But the gene alterations in spontaneous tumors are similar to constructed 3D organotypic tissues seeded with epithelial cells [140]. The formation of spheroids in fairly large diameter can result in a low oxygen concentration in the center. For chondrocytes, differentiation is stimulated by low oxygen, but for other cells types, hypoxia can cause necrosis in the spheroid core [141]. The different concentration gradients and the microenvironments (e.g., oxygen, nutrients, metabolites, paracrine factors, and growth factor) within the spheroid could investigate the mechanisms of tumor cell growth, differentiation, proliferation, and apoptosis as models of avascular tumors. Interactions between single cells and their surrounding matrix could also simulate cancer cell invasion in metastasis [142,143].

Cell patterns and vessel sprouting in 3D constructs are dependent on both cell aggregation and migration over time as required for angiogenesis and morphogenesis [144]. Maintaining the structural integrity and orientation of patterned microstructures is crucial in tissue regeneration, such as up to 2 weeks for cell spheroids [145]. Endothelial cells elongate morphology, decrease cell area, and form vascular networks in a matrix containing fibronectin, tenascin-C, collagen I, collagen IV, collagen VI, versican, and decorin [146]. Migration speed of mesenchymal, endothelial, and epithelial cancer cell spheroids is much faster than that of single cells and cell clusters [147]. Thus, monitoring cellular migration at the initial stage is critical in preparing cell spheroids. Cells patterned in an artificial matrix have different migration and aggregation behaviors, depending on the expression of cell interaction proteins (*i.e.*, cadherin) and cell-signaling molecules (*i.e.*, RhoA).

4D bio-printing is promising in fabricating the living tissues rapidly *in vitro* [148,149]. Cell spheroids make bio-printing tissues possible in the fourth dimension (time) because of their capabilities of rapid fusion, folding, and remodeling. One solution of developing functionally adaptive materials is to use external physiological stimuli to reprogram their shape, properties, and functionality [150]. Hydrogel-free bio-printing is attractive for its short fabrication time [15]. The integration of perfused capillaries inside the constructs is another milestone towards functional tissue and organ printing. MCSs are required to vascularize by themselves through biologically driven vasculogenesis or artificially developed submicron-sized vascular network. The significant tissue folding and remodeling in MCSs could enhance cell viability and preserve tissue functionality for a long time. Furthermore, the integration of macroscale vascular networks and their connection with capillaries are important for large-scale tissues and organ.

As the cells aggregate and self-assemble, cell-to-cell contact is maximized with the formation of numerous adhesions between surface adhesion molecules and even direct cell couplings such as gap junctions. MCSs mimic natural embryogenesis, morphogenesis, and organogenesis, exert biomechanical forces that change the shape, cytoskeleton, and function of the cells, and secrete biochemical signals that alter gene expression and cell function. They also synthesize, secrete, and assemble numerous ECM proteins. However, culturing spheroids is cumbersome and difficult to control. The signaling mechanisms involved in cell behavior of 3D constructs need to be completely understood. Surface adhesion molecules and the cytoskeletal network act together to control self-assembly and self-sorting, and the mechanical forces of cell-to-cell interactions play a role in these processes [151]. Cadherins are critical to self-assembly and self-sorting, high levels in the center of a transfected cells spheroid surrounded by cells with lower cadherin levels. Connexins (Cx43), a surface adhesion molecule, forms gap junctions for direct cell coupling and its adhesive function to self-assembly on par by cadherins [152]. Pannexin-1 (Panx1), another surface molecule with topographical similarities to connexins, forms channels that do not dock with other channels as pores leaking ATP, which activates purinergic receptors (P2X7) causing actin reorganization via elevated calcium levels. Drugs that target myosin-2 and actomyosin-dependent cell tension (Y-27632) block or slow the rate of self-assembly, alter self-sorting, and reduce tissue-specific activities [57,153]. Cell-cell adhesion and the formation of a stable 3D aggregate are enhanced by the recruitment of F-actin to the contacting regions to stabilize and strengthen intercellular adhesive interactions [153,154].

Multiple cell types with specific and essential biological functions to recapitulate the implanted tissue are necessary for vascularization, maintenance, and differentiation of stem cells. Current bio-printing involves either depositing multiple primary cells as the native tissue or proliferating and differentiating the stem cells into required cell types. Autologous cell sources could be obtained from biopsies or differentiation of autologous stem cells or through reprogramming in order to avoid the host rejection problem. However, most primary cells are hard to isolate and culture, and their short lifespan is inappropriate for the functionality in a long term. The use of stem cells in tissue engineering is promising because of their ability to proliferate in multipotent state and to generate multiple functional tissue-specific cell phenotypes. However, too little cell proliferation may result in the reduced viability of the implanted construct; whereas too much may produce hyperplasia or apoptosis. Initially, a high cellular proliferation rate may be desirable to populate the construct, but an appropriate value is preferred over the long term to achieve tissue homeostasis, self-renew, and respond to tissue damage or injury albeit without hyperplasia. The early cellular components of a developing tissue produce their own ECM components, appropriate cell signaling, and autonomous organization and pattern for the desired biological micro-architecture and function [155].

The biomechanical properties of normal human tissue and engineered tissue are partially dictated by the ECM organization [156], which is affected by cell-derived forces exerted on matrix components through intracellular tension generation because of cytoskeletal contractility [157]. HA is an essential high molecular weight biopolymer of cartilage ECM, providing a structural platform that binds large proteoglycan aggregates and chondrocytes and stimulating HA-binding proteins at the cell surface to produce an intercellular signal. The mechanical properties of normal cartilage tissue depend mostly on the structural integrity between the collagen network and the high concentration of sulfated GAGs. The enhanced compressive strength of the constructs by LIUS correlates with the accumulation of sulfated GAGs and collagen. Clinical LIUS exposure directly to the defect area after the injection of MSCs/fibrin-HA could lead to differentiation into cartilage [114].

Altogether, 3D bio-printing is promising in tissue engineering. The application of ultrasound in it, generating cell spheroids in USWF and enhancing tissue maturation by LIUS, may solve some challenges. The potential roles as well as the currently technical challenges of ultrasound in this emerging field are summarized in Table 2. With continuous research and technical improvement, this technology could move forward significantly and be accepted widely.

## Figures and Tables

**Table 1:** Comparison of bio-printing specifications.

Specification	Inkjet	Microextrusion	Laser Assisted	
Resolution	medium, 50 μm wide	medium-low, 5 μm–mm wide	high, μm wide	
Droplet size	50–300 μm	100–1000 μm	>20 μm	
Printing speed	fast (1–10,000 droplets/s)	slow (10–50 μm/s)	medium-fast (200–1600 mm/s)	
Materials	liquids, hydrogels	hydrogels, cell aggregates	cell in media	
Material viscosity	3.5–12 mPa/s	30–6 × 10^7^ mPa/s	1–300 mPa/s	
Cell density	low, <10^6^ cells/mL	High, cell spheroids	medium, 10^8^ cells/mL	
Multicellular feasibility	yes	yes	yes	
Preparation time	short	short-medium	long	
Mechanical integrity	low	high	low	
Fabrication time	long	long-medium	short	
Cell viability	high, >85%	medium-high, 40%–80%	medium, >95%	
Throughput	high	medium	low-medium	
Single-cell printing	low	medium	high	
Gelation speed	high	medium	high	
Printer price	low	medium	high	
Commercial availability	yes	yes	yes	
Advantages	affordable, versatile	multiple compositions, good mechanical properties	high accuracy, single cell manipulation, high-viscosity material	
Disadvantages	low viscosity, low strength	shear stress on nozzle tip, relatively low accuracy	cell unfriendly, low scalability, low viscosity in 3D build-up	

**Table 2:** Summary of the potential of ultrasound in 3D bioprinting.

Imaging	Sonography has much lower resolution than MRI and CT to illustrate the composition and structure of tissue clearly. But it can monitor the condition of bioprinted parts *in vivo* in real time at much lower cost.
Bioink preparation	USWF can generate various types of cell spheroids efficiently and quickly in a high cell viability using easy operation. Design of the device and optimization of operating parameters need specific knowledge, acoustics.
Tissue fusion	Acoustic field may be beneficial in enhancing the tissue fusion by the acoustic radiation force or mechanical vibration, which needs more experimental evidence.
Tissue maturation	LIUS could enhance the differentiation of stem cells effectively and highly compatible with the current bioreactors and tissue maturation approaches. Appropriate control of the release of growth factor at different stages of tissue maturation using different cell types is challenging.
